# Intranasal esketamine: real-world clinical practice in treatment-resistant depression and factors associated with treatment response

**DOI:** 10.1186/s12888-026-07980-8

**Published:** 2026-03-17

**Authors:** Bernhard T. Baune, Kevin Rosemann, Dimitri Hefter, Mustafa Tonkul, Erhan Kavakbasi

**Affiliations:** 1 Department of Psychiatry, University Hospital Münster, University of Münster, Albert-Schweitzer-Campus 1, Münster, 48149 Germany; 2 Department of Psychiatry, Melbourne Medical School, The University of Melbourne, Melbourne, Australia; 3 The Florey Institute of Neuroscience and Mental Health, The University of Melbourne, Parkville, VIC Australia; 4 Department of Psychiatry, Psychotherapy and Psychosomatics, RWTH Aachen University, Aachen, Germany

**Keywords:** Treatment resistant depression, Intranasal esketamine, Major depression, MDD, Novel antidepressant agents

## Abstract

**Background:**

Intranasal esketamine has recently emerged as an innovative treatment option for treatment-resistant depression (TRD). This retrospective, uncontrolled study aimed to evaluate the real-world effectiveness of esketamine in an naturalistic inpatient setting and explore demographic and clinical factors associated with treatment response.

**Methods:**

We conducted a chart review of 101 inpatients with TRD treated with intranasal esketamine at the University Hospital Münster, Germany. Depression severity was assessed pretreatment and posttreatment with the Montgomery-Åsberg Depression Rating Scale (MADRS) and Beck’s Depression Inventory-II (BDI-II). Repeated measures ANOVAs and logistic regression models were applied to identify treatment outcomes and associated factors.

**Results:**

Patients (mean age 47.7 years; 51.5% women) presented with severe depression and frequent psychiatric comorbidities. Esketamine treatment led to significant improvements in MADRS (mean reduction − 10.7, *p* < 0.001) and BDI-II scores (mean reduction − 11.5, *p* < 0.001), with large effect sizes. Suicidality scores decreased significantly as well. Overall, 28.8% achieved response, 52.5% at least partial response, and 19.3% remission. Older age was associated with higher remission likelihood (OR 4.06, *p* = 0.041), while male gender was associated with partial response (OR 3.71, *p* = 0.012). Extended induction beyond eight sessions was particularly beneficial for older patients and those with psychiatric comorbidities. No treatment-related serious adverse events were observed.

**Conclusions:**

In this large real-world inpatient cohort, intranasal esketamine significantly improved depression severity. Older patients and those with comorbid psychiatric disorders may particularly benefit from extended induction treatment. These findings support intranasal esketamine as an effective therapeutic option in TRD while highlighting the need for further controlled studies to refine patient selection and optimize treatment protocols.

**Clinical trial number:**

Not applicable.

## Background

Major depressive disorder (MDD) is a leading contributor to non-fatal health loss worldwide and a major driver of years lived with disability (YLDs). The Global Burden of Disease (GBD) 2019 analyses attribute ~ 15% of global YLDs to mental disorders, with depressive disorders among the highest-ranking causes across age groups and regions [1, 2]. Despite decades of therapeutic advances, a substantial subset of patients experience persistent symptoms and functional impairment despite adequate treatment exposure—commonly termed *treatment-resistant depression* (TRD). Remission probability declines with each successive pharmacotherapy step in routine practice, underscoring the clinical and societal consequences of treatment resistance [3]. Estimates of TRD prevalence vary with case definition and setting, but population-based and health-system cohorts suggest that roughly 10–30% of patients with major depressive disorder (MDD) meet TRD criteria at some point during their illness [4].

The limited speed and magnitude of response to conventional monoaminergic antidepressants have catalysed development of rapid-acting antidepressants (RAADs) targeting glutamatergic signalling. Mechanistically, ketamine-class agents rapidly enhance synaptic plasticity by modulating NMDA/AMPA throughput and neurotrophic signalling, offering relief within hours rather than weeks [5, 6]. Esketamine— the S-enantiomer of ketamine—was the first RAAD approved for TRD, initially as an adjunct to a newly initiated oral antidepressant (AD). By blocking NMDA receptors on GABAergic interneurons, esketamine leads to increased glutamate release, resulting in postsynaptic activation of AMPA receptors and subsequent synaptogenesis [7]. Across the phase 3 program in adult TRD, adding intranasal esketamine to an oral AD produced clinically meaningful symptom reductions by day 2 and sustained benefits through day 28 in short-term trials (e.g., TRANSFORM-2), with mixed results in specific populations (e.g., older adults in TRANSFORM-3) [8–10]. However, the TRANSFORM-1 trial failed to meet its primary endpoint, which was the change in depression severity at day 28 [9]. Longer-term maintenance data (SUSTAIN-1 relapse-prevention RCT and the SUSTAIN-2 open-label extension) showed that continued esketamine plus an oral AD reduced relapse risk and supported sustained effectiveness and safety over many months [11, 12]. In patients with MDD and acute suicidal ideation or behaviour (MDSI), the ASPIRE I/II trials demonstrated rapid improvement in depressive symptoms within 24 h when esketamine was added to comprehensive standard of care [13, 14]. Head-to-head evidence further indicates superiority of esketamine plus an oral AD over quetiapine XR-based strategies for achieving acute and sustained remission in TRD (ESCAPE-TRD) [15].

Regulatory guidance has continued to evolve with accumulating evidence. As of January 2025, the U.S. prescribing information indicates esketamine for TRD as monotherapy or in conjunction with an oral AD, and for depressive symptoms in MDD with acute suicidal ideation or behaviour only in conjunction with an oral AD. The label also emphasizes REMS-program (risk evaluation and mitigation strategy) administration with monitoring for sedation, dissociation, blood-pressure elevations, and clarifies that effectiveness in preventing suicide has not been demonstrated [16].

While randomized controlled trials (RCTs) establish efficacy and safety, their stringent eligibility criteria limit generalizability to the broader TRD population—who frequently present with psychiatric and medical comorbidity, polypharmacy, variable illness chronicity, and complex care pathways. This study aims to bridge the gap between randomized controlled trials and real-world clinical practice by providing data from a relatively large real-world cohort. Such data from real-world populations are necessary to translate findings from RCTs to routine clinical settings. Large registry- and claims-based cohorts highlight the high clinical and economic burden associated with TRD and the frequency of delayed or non-sequential treatment changes in usual care [17]. Real-world evidence (RWE), as presented in this study, therefore provides essential complementary data, characterizing treatment patterns, effectiveness, and tolerability in more heterogeneous. Emerging RWE on intranasal esketamine—spanning retrospective clinic cohorts and multicenter national programs—has generally found clinically meaningful symptom reductions and acceptable tolerability under routine conditions, while illuminating practice-relevant questions such as optimal induction duration, dose optimization, adherence, and management of comorbidities [18–21].

### Objectives of the present study

To close the gap between manufacturer-initiated studies and real-world clinical practice, in this study we sought to extend the RWE base by analyzing outcomes of intranasal esketamine administered in routine clinical practice. Specifically, our objectives were to: (i) evaluate real-world clinical effectiveness (change in MADRS and BDI-II, including suicidality indices) under naturalistic conditions in patients with high number of comorbid psychiatric and medical conditions and concomitant treatments; (ii) examine the impact of clinical parameters (e.g., age, sex, comorbidities, prior treatments) on antidepressant response; (iii) describe the clinical and demographic characteristics of esketamine-treated patients; and (iv) estimate response, partial response, and remission rates and explore factors associated with each. By focusing on a naturalistic cohort, this work aims to inform clinical decision-making on patient selection, dosing/optimization (including extended induction when used), and expectation-setting in the translation of esketamine into everyday TRD care.

## Methods

### Study desing and data collection

This is a retrospective chart review of patients treated with intranasal esketamine at the Department of Psychiatry, University Hospital Münster Germany. We retrospectively collected data on patients who were treated with intranasal esketamine and were rated for depression severity as part of routine clinical practice. All patients admitted to hospital due to clinical diagnosis of unipolar treatment-resistant depression (TRD) who received inpatient psychiatric treatment with intranasal esketamine have been included in this study. The diagnosis of major depression as well as treatment resistance was assessed and confirmed during an unstructured clinical interview conducted by the treatment team, based on ICD-10 criteria, without the use of a structured diagnostic instrument. Treatment-resistant depression was considered if patients did not sufficiently respond to at least two prior antidepressant trials in adequate dose and duration. Adequate dose refers to the minimum effective dose according to the drug’s prescribing information. Adequate treatment duration is generally considered to be at least 4 weeks of treatment at an adequate dose. The TRD diagnosis has either been confirmed in patients interview by self-or by review of previous treatment reports.

The study has been approved by the local institutional review board of the University Hospital Münster (2023-435-f-S) and has been conducted following ethical principles stated in the Declaration of Helsinki. All data have been collected from patients’ medical records and analyzed using the software IBM SPSS Statistics, Version 29, Armonk, New York, USA. Due to the regulations of the North Rhine-Westphalian health data protection law, written informed consent was not required to collect and analyze routine clinical data that clinicians could access as part of their clinical duties.

Depression severity has been assessed as part of routine clinical practice using the investigator-rated MADRS (Montgomery-Åsberg Depression Rating Scale) and the patient-reported Beck’s Depression Inventory (BDI-II) [22, 23]. For the assessment of suicidal ideations, we used the item 10 of the MADRS scale. The ratings took place during routine clinical treatment of the patients by the treating physicians and psychologist, who were not blinded. The assessments have been performed prior to treatment initiation as well as at the end of the induction treatment in an inpatient setting. In a subset of patients (*n* = 36) symptom ratings were also available after the first eight treatment sessions, which corresponds to one month follow-up period after treatment initiation. Response was defined as a decrease of at least 50% in the MADRS score from baseline to post-treatment, whereas an improvement 30–50% in MADRS score has been considered as partial response. Remission was defined as a MADRS score of 10 or less at the follow-up evaluation.

### Esketamine treatment procedure

The FDA-approved (U. S. Food and Drug Administration) esketamine intranasal spray (Spravato, Johnson & Johnson, New Brunswick, New Jersey, USA) has been used for the esketamine treatment. Esketamine is provided in nasal applicators each containing 28 mg of esketamine hydrochloride. Depending on the dose chosen, 1–3 dose applications (28–84 mg esketamine) have been used in a single session. The decision to initiate esketamine treatment as well as the dose selection and adjustment have been made by a senior psychiatrist with expertise in the management of treatment-resistant depression. In patients with treatment-resistant depression, esketamine is usually initiated at 56 mg, with the dose increased to 84 mg in the subsequent sessions. In elderly patients over the age of 65, the starting dose is 28 mg with subsequent dose increase [16].

In the European Union, esketamine is approved for the management of treatment-resistant depression as well as for the therapy of psychiatric emergencies in patients with major depression. After the decision for esketamine treatment has been made, patients received extensive information and gave informed consent for esketamine treatment. Contraindication have been ruled out and laboratory test, electrocardiogram and blood pressure have been checked before treatment initiation. Patients usually received twice weekly session for one months (8 sessions). This induction period could be extended, if patients showed improvement, but failed to achieve remission. The decision to extend the induction treatment or to cease esketamine has been determined by the treating senior psychiatrist based on clinical judgment in a shared decision-making process with the patients. The esketamine treatment sessions took place in a quiet and darkened room providing capacity for up to five simultaneous esketamine treatments. Patients were observed and monitored by a nurse during the treatment sessions. Blood pressure has been measured before and 40 min after esketamine application.

### Statistics

To describe demographic data, we used descriptive statistics. We used continuous variables (age, number of psychotropic agents, number of esketamine treatment sessions, pretreatment MADRS and BDI-II scores) and categorical variables (psychiatric comorbidity (yes/no), history of ECT, class of antidepressant medication) to describe the demographic characteristics of the sample.

The change in mean MADRS and BDI-II as well as the calculation of effect sizes has been performed using a one-sided, paired t-test [24]. We chose Cohen’s d as effect size measure [25]. Pretreatment and posttreatment BDI-II and MADRS scores were normally distributed. To identify the impact of different between-subject factors we used a repeated measured analysis of variance (ANOVA) with MADRS pretreatment and post-treatment as within-subject factor and the following parameters as between-subject factors: gender (men/women); age (< 60 or 60 and older); lifetime history of electroconvulsive therapy (yes/no); psychiatric comorbidity (yes/no). The associations between the between-subject factors were not significant, suggesting that multicollinearity was not present. We focused on the main effects of the between-subject factors rather than on their interactions. Given the sample size, the study was likely underpowered to reliably detect interaction effects of the between-subject factors. Additionally, we included the number of esketamine treatment sessions as a confounding covariate in the statistical model. We also performed a second repeated measures ANOVA with the BDI-II score pretreatment and post-treatment as within-subject factor. In the ANOVA models with two time points, sphericity is fulfilled by definition. We also conducted an additional repeated measures ANOVA and analysis of the change in MADRS score over three timepoints (baseline, after the eighth session, follow-up) to evaluate the effects of extended induction treatment. In this model, sphericity was met (Maulchy W = 0.865, *p* = 0.123). The impact of the categorial variables (gender, age, psychiatric comorbidity and lifetime history of ECT) on response, partial response and remission rates as well as the calculation of odds ratios for response, partial response and remission has been accomplished using binary logistic regression models with response, partial response and remission as dependent variables.

## Results

### Sample characteristics

We analysed 101 inpatients with treatment-resistant unipolar depression (mean age 47.7 years; 51.5% women). Psychotic features were present in 7.9%. These patients had typical major depression with mood-related psychotic features but did not have bizarre delusions as seen in schizophrenia. Psychiatric comorbidity was common (56.4% of the patients had at least one comorbid psychiatric disorder; most frequently personality disorders (15.8%), posttraumatic stress disorder (PTSD, 13.9%), and substance use disorders (12.9%). Furthermore, approximately 50% had at least one medical comorbidity. Indication for esketamine was standard TRD in 81.2% and a psychiatric emergency in 18.8%. Baseline depression severity was high (MADRS 32.8; BDI-II 37.7); mean MADRS suicidality item at baseline was 2.4. See Table 1 for subgroup comparisons by age and gender.

**Table 1:** Demographic data. Distribution of demographic characteristics by age and gender. Number of agents and MADRS item 10 were not normally distributed, thus we conducted the Mann-Whitney-U Test, whereas for other continuous variables a two-sided t-test was applied

		Age (< 60 years) (*n* = 75)	Age (≥ 60 years) (*n* = 26)	*p*-value	Women (*n* = 52)	Men (*n* = 49)	*p*-value
Psychiatric comorbidity (%)		69.3	19.2	< 0.001	61.5	51.0	0.320
Lifetime history of ECT (%)		48.0	65.4	0.172	48.1	57.1	0.427
Medication	SSRI (%)	21.3	19.2	0.516	23.1	18.4	0.710
	SNRI (%)	62.7	73.1		65.4	65.3	
	Other (%)	16.0	7.7		11.5	16.3	
Number of agents (n)		3.1	3.0	0.675	3.2	3.0	0.610
Number of esketamine sessions (n)		10.4	11.8	0.165	10.7	10.9	0.804
MADRS pretre. (mean) (± SD)		32.7 (± 8.5)	32.1 (± 7.3)	0.717	33.6 (± 8.2)	31.3 (± 7.9)	0.188
BDI-II pretre. (mean) (± SD)		38.8 (± 10.9)	34.4 (± 9.0)	0.072	40.5 (± 9.9)	33.9 (± 10.3)	0.003
MADRS item 10 pretre. (mean) (± SD)		2.6 (± 1.5)	2.0 (± 1.2)	0.077	2.5 (± 1.4)	2.3 (± 1.4)	0.462

For categorial data we used the Chi-quadrate test. Pretre: pretreatment. ECT: electroconvulsive therapy. SD: standard deviation.

### Treatment exposure and concomitants

Patients received twice-weekly intranasal esketamine for a mean of 10.8 sessions; most initiated at 56 or 84 mg, with 92.1% receiving 84 mg at the final session. Background pharmacotherapy typically included a selective serotonin norepinephrine reuptake inhibitor (SNRI) or selective serotonin reuptake inhibitor (SSRI) (mean 3.1 psychotropic agents). Prior ECT (electroconvulsive therapy) was documented in 52.5%; small subsets had VNS (5.9%, vagus nerve stimulation), concurrent iTBS (*n* = 6, intermittent theta burst stimulation), or prior IV esketamine (13.9%).

### Primary outcomes

**Fig. 1:**
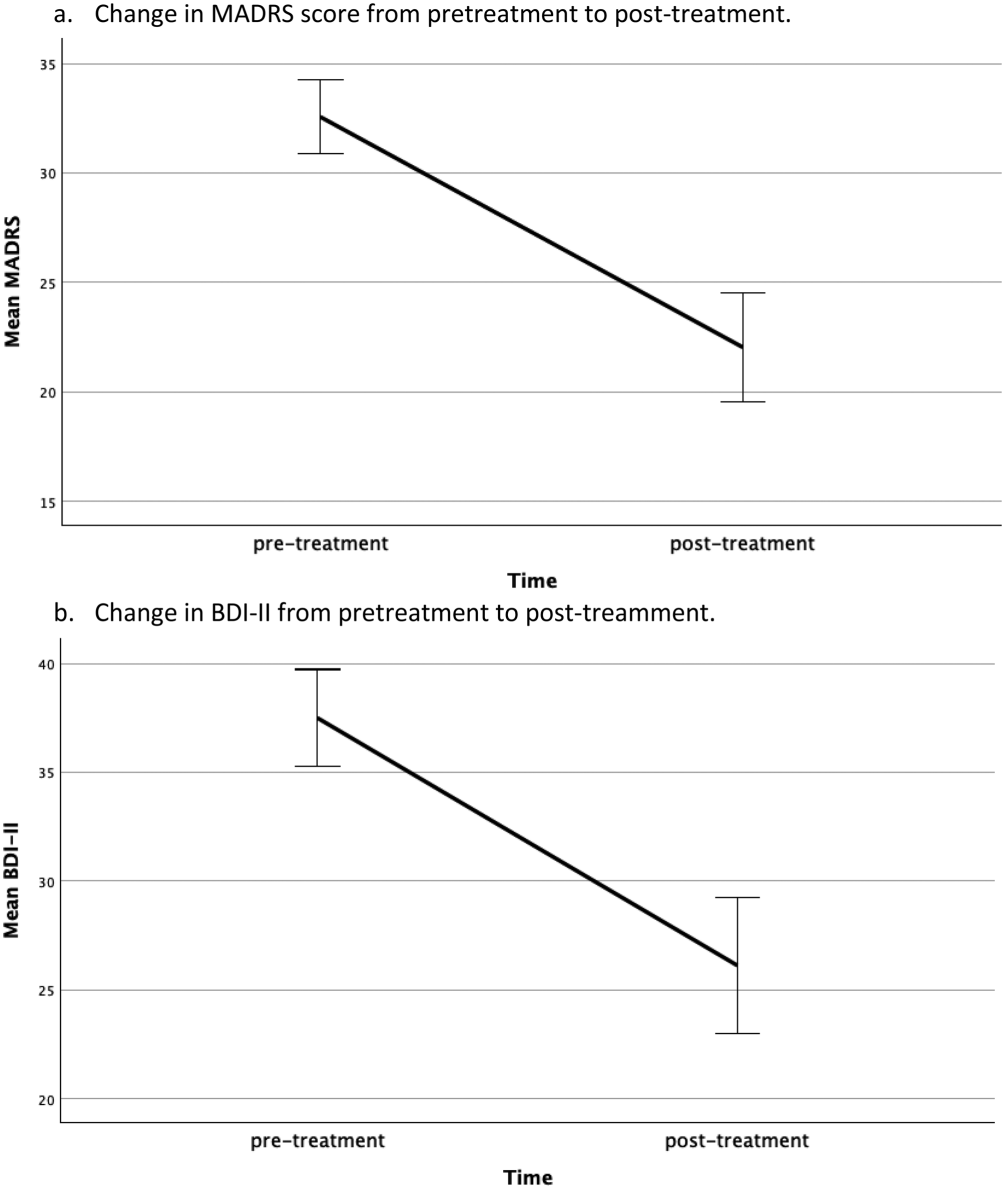
Change in main outcome parameters**.** Bars indicate 95%-confidence intervals

From pre- to post-series, clinician-rated and self-reported depres2antly: MADRS decreased by 10.7 points (95% CI (confidence interval) 8.3–13.0) and BDI-II by 11.5 points (95% CI 9.2–13.9), both with large effect sizes (Table 2). The MADRS suicidality item fell from 2.5 to 1.6 (mean change − 0.9; *p* < 0.001). Overall, 28.8% achieved response (≥ 50% MADRS reduction), 52.5% achieved at least partial response (≥ 30–<50% improvement), and 19.3% met remission (MADRS ≤ 10). Figure 1 illustrates pre-/post-changes.

**Table 2:** Esketamine treatment led to significant improvement in the main outcome measures with large effect sizes

	Baseline (mean) (±SD)	End of treatment	Reduction (mean) (±SD) (95%-CI)	T	Cohen’s d	p-value
MADRS	32.8 (±8.3)	22.1 (±11.4)	10.7 (±10.5) (8.3-13.0)	9.110	1.019	<0.001
BDI-II	37.7 (±10.8)	26.2 (±14.5)	11.5 (±10.6) (9.2-13.9)	9.697	1.084	<0.001

### Change over time and subgroup effects

**Fig. 2:**
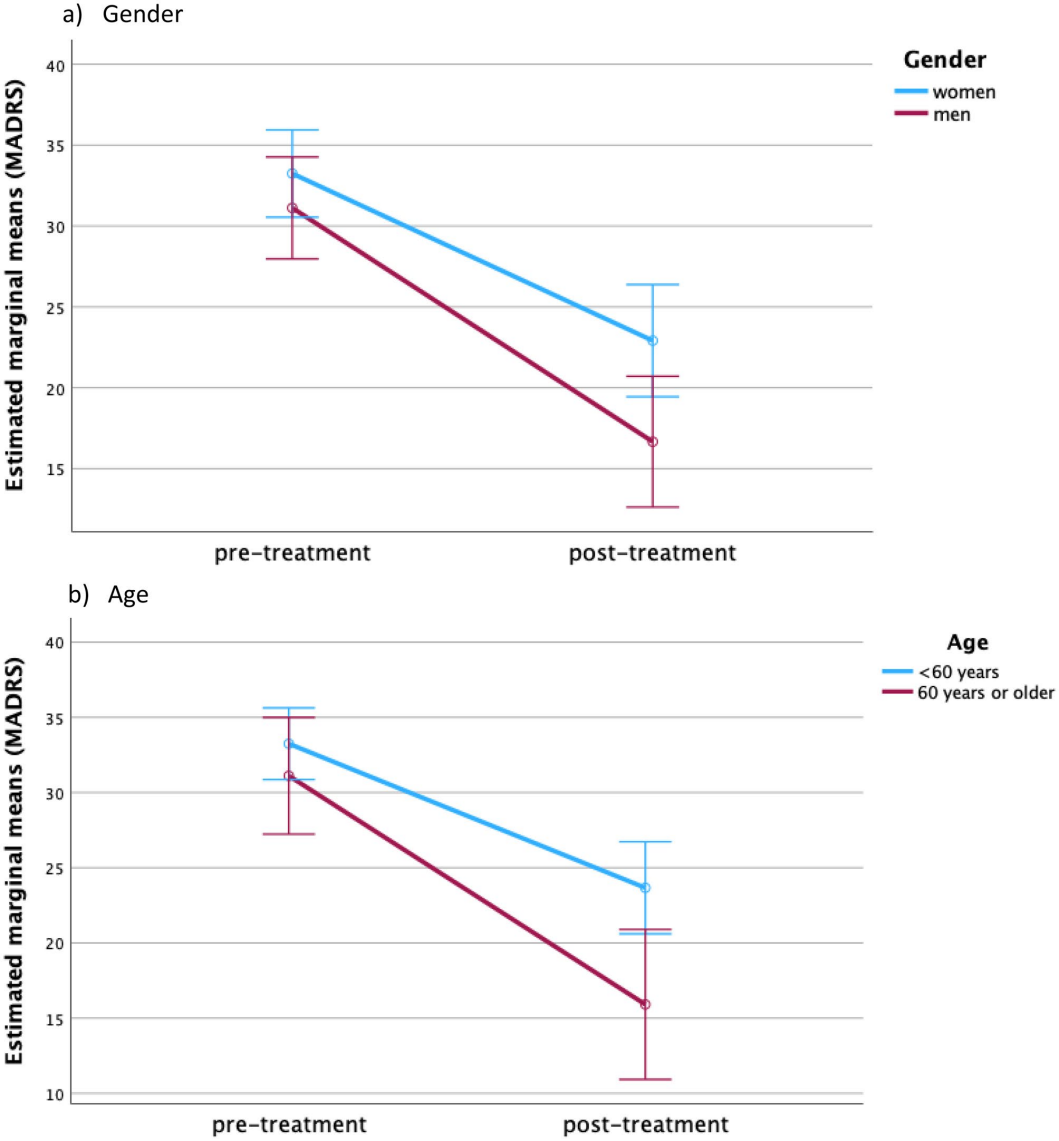

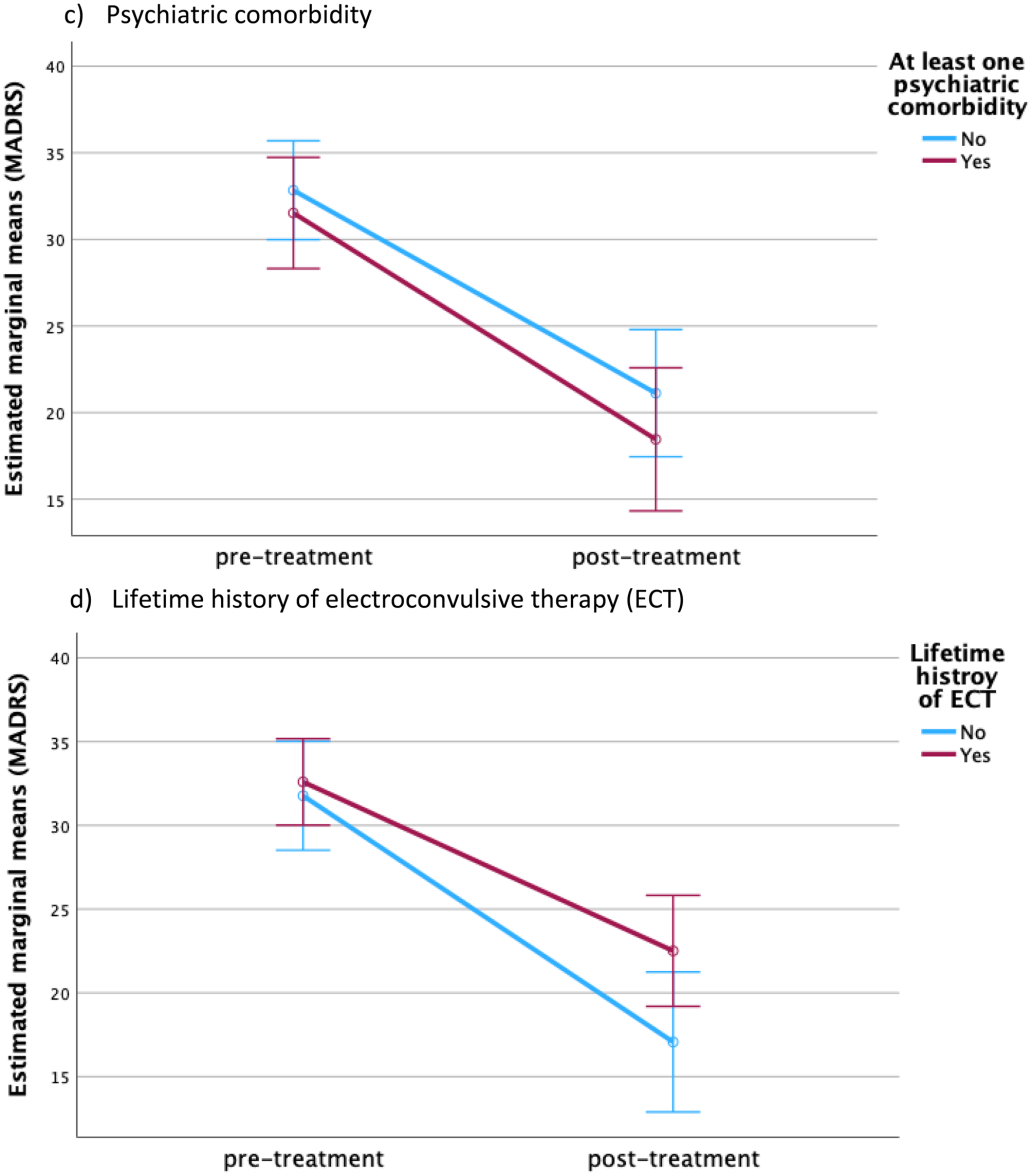
Change in MADRS score depending on between-subject factors. Graphs demonstrate change in MADRS score depending on between-subject factors over time. Bars indicate 95%-Confidence intervals

Repeated-measures ANOVA confirmed a significant effect of time on MADRS improvement without significant time×factor interactions; depression severity was higher in women and in patients < 60 years (Table 3; Fig. 2). For BDI-II, time effects were significant and interacted with age, indicating greater self-rated improvement in older patients.

**Table 3:** Repeated measures ANOVA. To investigate the impact of several between-subject factors on outcome measures and theirs change over time

Between-subject factor	Between-subject main effect		Between-subject interaction effect (time*between subject factor)		Between-subject main effect		Between-subject interaction effect (time*between subject factor)	
	MADRS				BDI-II			
	F	p-value	F	p-value	F	p-value	F	p-value
Gender	4.688	0.034	2.952	0.090	8.057	0.006	0.154	0.696
Age	4.479	0.038	3.761	0.056	7.108	0.009	4.069	0.047
Psychiatric comorbidity	0.905	0.345	0.273	0.603	0.013	0.911	0.042	0.837
Lifetime history of ECT	2.605	0.111	3.668	0.059	2.477	0.120	3.210	0.081
Number of esketamine sessions	0.875	0.353	0.558	0.457	0.607	0.438	2.402	0.125

Time from baseline to end of treatment as a within-subject factor had a significant effect on MADRS changes demonstrating overall significant improvement (F = 13.696, *p* < 0.001). Time from baseline to end of treatment as a within-subject factor had also a significant effect on BDI-II change indicating significant improvement self-rated depression severity (F = 19.916, *p* < 0.001). Furthermore, there was a significant interaction effect of time*age indicating greater improvement in BDI-II in patients of older age

### Factors associated with treatment outcome

In logistic models, no covariate significantly predicted *response*. Male gender predicted *partial response* (Odds Ration, OR 3.705; 95% CI 1.335–10.284; *p* = 0.012). Age ≥ 60 years predicted *remission* (OR 4.060; 95% CI 1.062–15.530; *p* = 0.041). The small number of patients over the age of 60 (*n* = 26) and the wide confidence intervals indicate uncertainty in the estimates. The effect of other variables was not significant (Table 4).

**Table 4:** Binary logistic regression model

Categorial variable	Regressions coefficient B	*p*-value	Odds Ratio	95% CI for Odds Ratio	Regressions coefficient B	*p*-value	Odds Ratio	95% CI for Odds Ratio	Regressions coefficient B	*p*-value	Odds Ratio	95% CI for Odds Ratio
Outcome	Response				Partial Response				Remission			
Gender (men)	0.069	0.894	1.072	0.385–2.982	1.310	0.012	3.705	1.335–10.284	0.667	0.268	1.949	0.599–6.340
Age (60 years or older)	0.870	0.152	2.387	0.725–7.859	1.066	0.081	2.903	0.876–9.621	1.401	0.041	4.060	1.062–15.530
Psychiatric comorbidity (yes)	0.114	0.844	1.120	0.362–3.463	0.965	0.086	2.626	0.873–7.894	0.012	0.986	1.012	0.273–3.747
Lifetime history of ECT (yes)	-0.699	0.183	0.497	0.178–1.392	-0.976	0.058	0.377	0.137–1.034	-0.700	0.254	0.497	0.149–1.654

The impact of the categorial variables gender, age (<60 vs. 60 years and older), psychiatric comorbidity (yes/no) and lifetime history of ECT (no/yes) on MADRS response, partial response and remission rates has been investigated using a binary logistic regression model. The odds ratio for men to achieve partial response was 3.705 compared to women. Regarding remission there was a significant impact of age. The odds ratio for patients of older age (60 years or older) to fulfill remission criteria was 4.060 compared to younger patients

### Extended induction (exploratory)

**Fig. 3:**
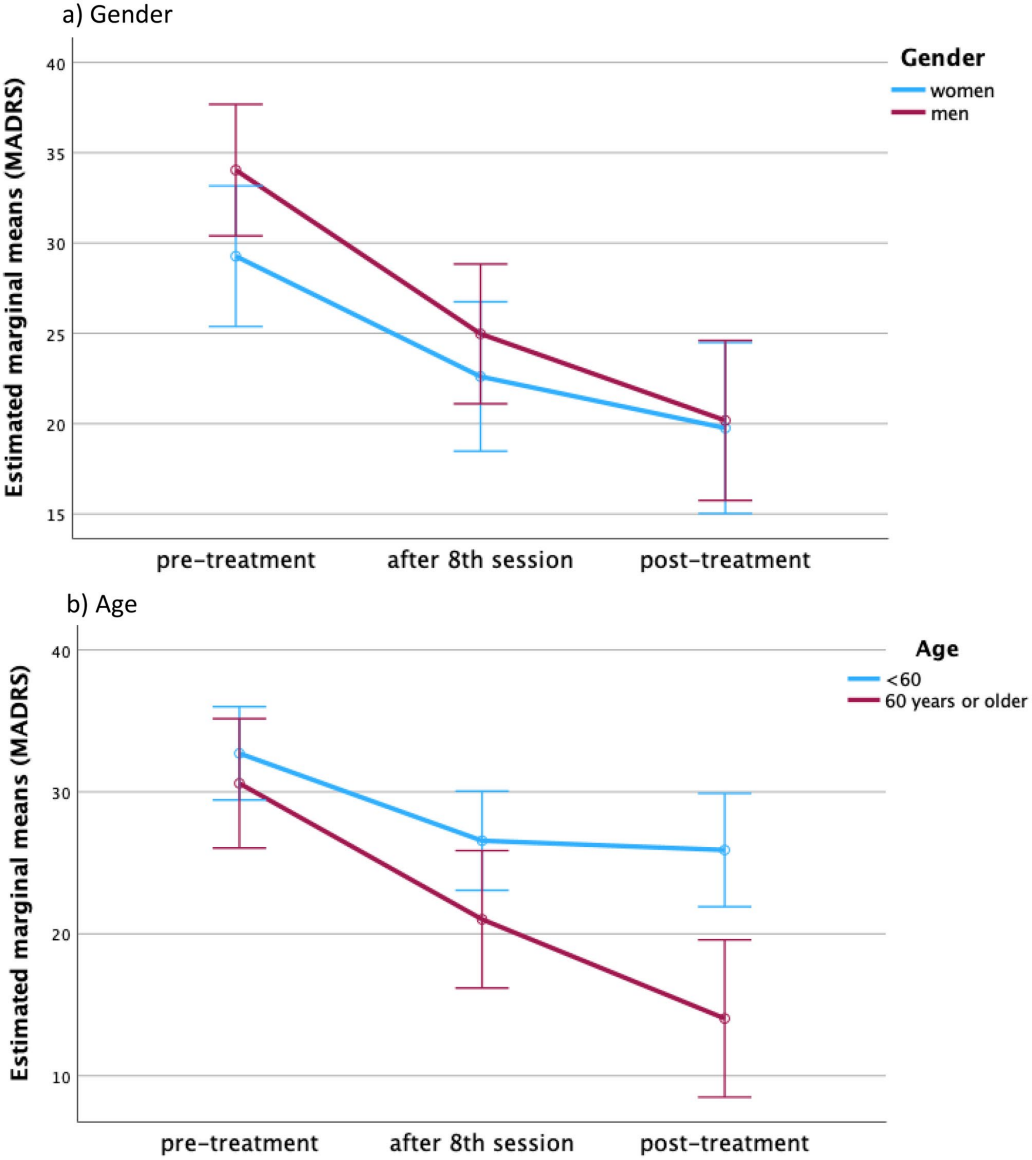

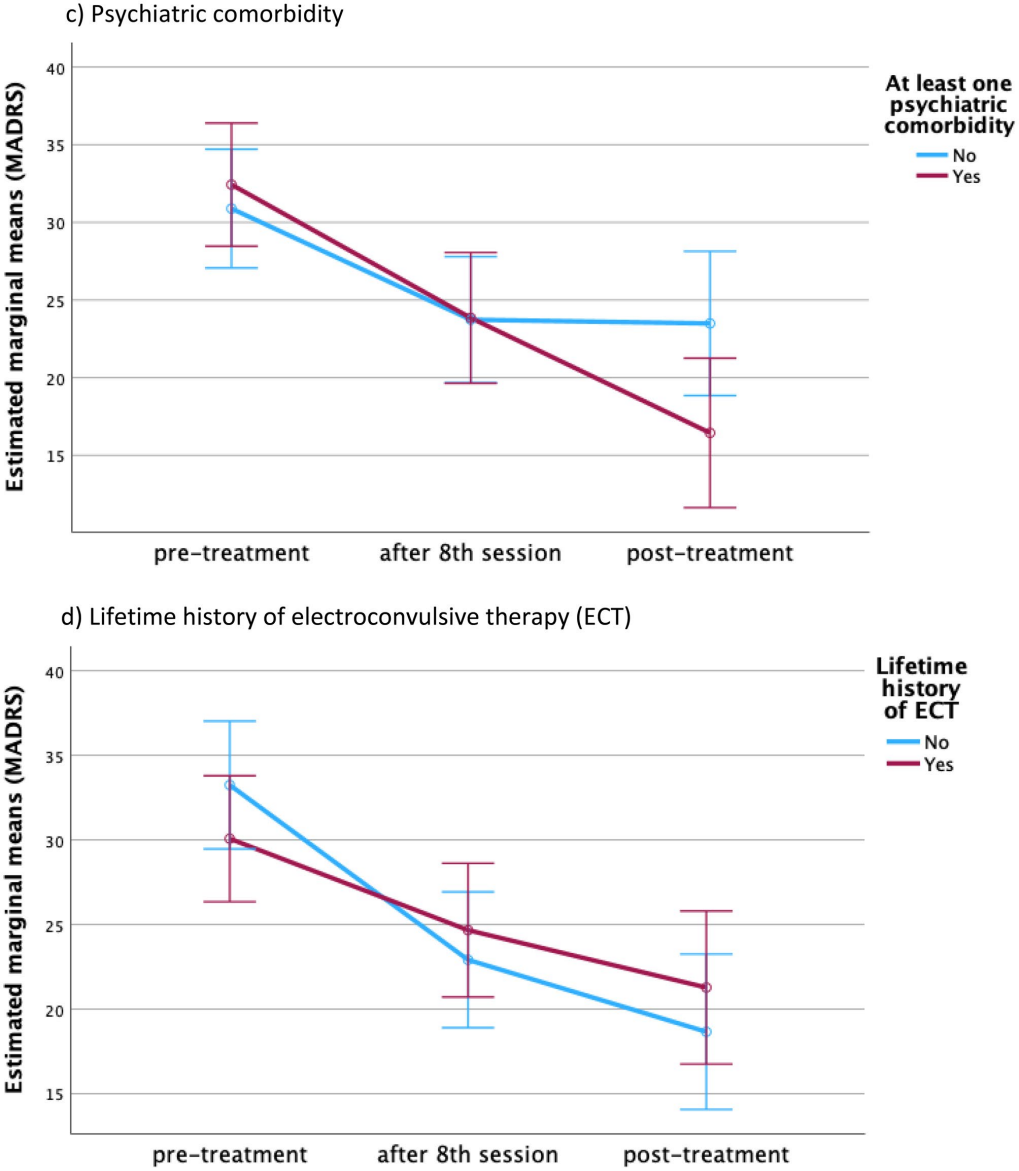
Extended induction treatment**.** The repeated measures ANOVA revealed that patients of older age (60 years or older, F = 5.861, *p* = 0.005) as well as patients with at least one psychiatric comorbidity (F = 5.476, *p* = 0.007) were most likely to benefit from extending the induction period beyond the eighth sessions, whereas patients of younger age (< 60) as well as patients without a psychiatric comorbidity did not show further improvement beyond the eighth session. Lifetime history of electroconvulsive therapy as well as gender did not impact the effect of treatment extension significantly

Among patients with ratings at baseline, post-session 8, and end of treatment (*n* = 36), an extended induction beyond eight sessions significantly benefitted older adults (≥ 60 years) and those with psychiatric comorbidity. Patients < 60 years and those without comorbidity showed little additional gain. Lifetime ECT and gender did not modify extension effects (Fig. 3).

### Induction and maintenance treatment

The mean duration of the induction treatment period was 6.6 weeks (median 6 weeks). The induction treatment was completely performed as an inpatient procedure. About 54.1% of the patients have been planned for a maintenance treatment regimen. The mean duration of the treatment intervals in the maintenance phase was 4.9 weeks (median 4). The mean duration of the maintenance treatment phase was 30.3 weeks (median 17.5). In 72.7% esketamine treatment had been terminated at time of analysis, whereas the other patients were still in the maintenance treatment phase. The mean total number of esketamine sessions was 23.5 (median, 20, induction plus maintenance period).

### Safety

No treatment-related serious adverse events occurred. Premature discontinuation occurred in 8.9% (most < 6 sessions), commonly due to dissociation (*n* = 4) or sedation (*n* = 1); two patients switched for non-response, two left inpatient care early. One non-fatal suicide attempt was recorded.

## Discussion

In this large, real-world inpatient cohort with treatment-resistant depression (TRD), intranasal esketamine was associated with significant improvements in clinician-rated and self-reported depressive symptoms, alongside a significant reduction in suicidal ideation. At the series endpoint, mean MADRS and BDI-II scores decreased by ~ 11 points, respectively; 28.8% achieved response, 52.5% at least partial response, and 19.3% remission. Notably, older age was independently associated with remission (OR 4.06), whereas male sex had higher odds of partial response (OR 3.71). However, the large confidence intervals for the odd ratios indicate uncertainty in the results. An exploratory analysis suggested that extending the induction beyond eight sessions was especially beneficial in older patients and in those with psychiatric comorbidity. No treatment-related serious adverse events (AEs) were observed, except for one non-fatal suicide attempt; discontinuations (8.9%) were mainly due to dissociation or sedation. These results support intranasal esketamine as a pragmatic treatment option in complex, comorbid TRD seen on inpatient units and refine hypotheses about which patients may benefit from an extended acute phase.

### Contextualization with randomized evidence

Our findings align with the pivotal phase 3 program. In acutely ill TRD outpatients, esketamine plus a newly initiated oral antidepressant (AD) produced rapid, clinically relevant improvements over AD plus placebo nasal spray in TRANSFORM-2 and supportive benefits in TRANSFORM-1. The latter study failed to demonstrate the superiority of esketamine over placebo for the primary endpoint [9]. Although the elderly-only TRANSFORM-3 trial narrowly missed its primary endpoint overall, signal was stronger in 65–74-year-olds, and patients who continued into open-label treatment improved further [10]. For maintenance, SUSTAIN-1 demonstrated that continuing esketamine plus AD reduced relapse risk versus AD plus placebo among patients who achieved response or remission during induction [11]. Longer-term, the open-label SUSTAIN-2 program showed sustained benefit with individualized maintenance dosing up to one year [12]. Most recently, the head-to-head ESCAPE-TRD trial showed esketamine + SSRI/SNRI was superior to quetiapine XR augmentation + SSRI/SNRI for remission at week 8 and for remaining relapse-free to week 32, reinforcing clinical effectiveness against an active, guideline-supported comparator [15]. Notably, the response and remission rates in our real-world study were lower than those reported in the manufacturer-initiated trials [15].

### Consistency with real-world effectiveness

Rates of response and remission in our inpatient sample fall within the range reported in observational cohorts that include patients with high comorbidity, prior neuromodulation, and complex polypharmacy. The multicenter REAL-ESK study (*N* = 116) reported significant improvements with acceptable tolerability across diagnostic and comorbidity subgroups [19]. In the French ESKALE study (*N* = 157, 12-month follow-up), ~ 40% achieved response and ~ 20% remission at one month in still-treated patients; discontinuation rates were substantial (79.6%), reflecting routine-care dynamics and stringent monitoring requirements [20]. The main reasons for esketamine discontinuation in the ESKALE study were insufficient symptom improvement (52.0%), regulatory requirement of the prescribing information (26.4%), patients preference (24.8%) and adverse events (8.8%) such as increased blood pressure, sedation and dissociation [20]. A U.S. retrospective cohort similarly found meaningful symptom reductions with an acceptable safety profile [18]. Together with these reports, our results add inpatient-setting data from a relatively large, systematically characterized cohort.

### Patient factors and treatment tailoring

Two findings—greater odds of remission with older age and a signal for partial response in men—deserve comment. Post-hoc analyses of SUSTAIN-2 showed broadly comparable antidepressant benefit and safety in older (≥ 65 years) and younger adults, suggesting that age alone should not deter use when cardiovascular and cognitive safety are monitored [26]. Our observation that extended induction favored older adults and those with psychiatric comorbidity dovetails with phase-3 data indicating that treatment frequency and duration should be individualized during optimization/maintenance to sustain or deepen benefit [27]. These convergent data support a pragmatic approach: evaluate benefit at the end of the 4-week induction per labeling, and for partial responders—particularly older patients or those with comorbidity—consider extending the acute phase before transitioning to less-frequent maintenance.

### Symptom domains and suicidality

We observed a significant reduction in the MADRS suicidality item. In MDD patients with acute suicidal ideation or behavior, the ASPIRE I and ASPIRE II trials demonstrated rapid reductions in overall depressive symptoms with esketamine plus comprehensive standard of care; however, neither trial established a specific anti-suicidal effect independent of overall mood improvement [13, 14]. Consistent with regulatory labeling, the effectiveness of esketamine in preventing suicide or reducing suicidal ideation/behavior has not been demonstrated, and hospitalization remains indicated when clinically warranted [16]. Clinicians should interpret improvements in item-level suicidality as encouraging but continue full risk management per guidelines.

### Safety and tolerability

Our discontinuations were primarily due to dissociation and sedation, well-characterized AEs that are generally transient and dose-session–linked. The U.S. Prescribing Information highlights boxed warnings for sedation, dissociation, respiratory depression, and misuse/abuse risk and mandates REMS-based observation with blood pressure and respiratory monitoring [16]. Large post-approval pharmacovigilance analyses report respiratory depression to be rare (≈ 1 in 20,000 sessions) and typically manageable with supportive care during monitoring [28]. In long-term follow-up (SUSTAIN-3 interim), no new safety signals were observed up to ~ 4.5 years of intermittent treatment, and urinary adverse events did not suggest cystitis risk above background rates; sedation and dissociation remained mostly same-day and self-limited [29]. Our absence of treatment-related serious AEs aligns with these data, though AE were not systematically assessed in this study and AE ascertainment was limited in routine care. We observed one non-fatal suicide attempt, indicating that suicidal ideations should be regularly monitored during esketamine treatment series.

### Clinical implications

Several pragmatic points arise. First, inpatient initiation could be leveraged to titrate to 84 mg in most patients and to engage in shared decision-making about extending induction when improvement is present but sub-threshold for remission. Second, comorbidity should not preclude esketamine; our data and prior reports suggest that real-world complexity does not necessarily blunt effectiveness [18–20]. Third, because labeling now permits use for TRD as monotherapy or with an oral AD in adults, clinicians can tailor background pharmacotherapy to prior tolerability and patient preference while maintaining required monitoring [16].

### Strengths and limitations

Strengths include one of the largest inpatient, real-world cohorts to date; inclusion of patients with substantial comorbidity; and analyses of factors associated with treatment response and extended induction—issues under-represented in trials. Limitations include the retrospective and naturalistic study design, absence of a control group and randomization and lack of blinded raters, allowance of potentially confounding concomitant treatment changes, and incomplete mid-induction assessments. Furthermore, adverse events were not systematically assessed in this study. Moreover, concomitant treatments may have influences treatment outcomes. The lack of blinded raters may have introduced expectancy bias. Incomplete mid-induction assessments mean that data on extended induction treatment are considered exploratory. These constraints limit causal inference and precision around effect sizes, particularly for suicidality outcomes and tolerability estimates. The relatively small sample size carries the risk of overfitting. Performing numerous subgroup analyses increases the risk of Type I errors. As no formal correction was applied, the results should be interpreted cautiously. Nevertheless, the direction and magnitude of change are consistent with randomized and real-world data, and the findings on factors associated with treatment response generate testable hypotheses for prospective work.

## Conclusions

In routine inpatient practice, intranasal esketamine was associated with improvement in depression severity and reduced suicidality scores, with tolerability consistent with known risks. Older adults and patients with psychiatric comorbidity may particularly benefit from extending the acute phase beyond eight sessions, and male sex is associated with partial improvement. Prospective, controlled studies should confirm which clinical profiles warrant extended induction and clarify strategies to convert partial response into remission while optimizing long-term safety monitoring.

## Data Availability

The datasets analysed during the current study are not publicly available due to local regulations, but are available from the corresponding author on reasonable request.

## Abbreviations

MDD: Major depressive disorder
YLD: Years lived with disability
GBD: Global Burden of Disease
TRD: Treatment-resistant depression
MADRS: Montgomery-Åsberg Depression Rating Scale
BDI-II: Beck’s Depression Inventory-II
ANOVA: Analysis of variance
OR: Odds ratio
RAAD: Rapid acting antidepressants
NMDA: N-methyl-D-aspartate
AMPA: Alpha-amino-3-hydroxy-5-methyl-4-isoxazolepropionic acid
GABA: Gamma aminobutyric acid
AD: Antidepressant
AE: Adverse event
RCT: Randomized-controlled trial
REMS: Risk evaluation and mitigation strategy
RWE: Real world evidence
SD: Standard deviation
FDA: Food and Drug Administration
ECT: Electroconvulsive therapy
VNS: Vagus nerve stimulation
iTBS: Intermittent theta burst stimulation
XR: Extended release
